# Remote control of resistive switching in TiO_2_ based resistive random access memory device

**DOI:** 10.1038/s41598-017-17607-4

**Published:** 2017-12-08

**Authors:** Dwipak Prasad Sahu, S. Narayana Jammalamadaka

**Affiliations:** Magnetic Materials and Device Physics Laboratory, Department of Physics, Indian Institute of Technology Hyderabad, Hyderabad, 502 285 India

## Abstract

We report on the magnetic field control of a bipolar resistive switching in Ag/TiO_2_/FTO based resistive random access memory device through I–V characteristics. Essentially, in the presence of magnetic field and in the low resistance state, an abrupt change in the resistance of the device demands higher voltage, hinting that residual Lorentz force plays a significant role in controlling the resistance state. Endurance characteristics of the device infer that there is no degradation of the device even after repeated cycling, which ensures that the switching of resistance between ‘off’ and ‘on’ states is reproducible, reversible and controllable. Magnetic field control of ‘on’ and ‘off’ states in endurance characteristics suggest that this device can be controlled in a remote way for multi-bit data storage.

## Introduction

Non-volatile memory (NVM) technology indeed requires intensive research as the conventional silicon (Si) based memories are approaching to their scaling limits. In particular, among various promising modern NVM technologies, resistive random access memory (RRAM) has attracted a great deal of scientific and technological interest owing to its easy fabrication, high density and promising performances. RRAM devices would work based on the resistive switching (RS) phenomena in which the resistance state can be altered between high resistance state (HRS) and low resistance state (LRS) by controlling applied voltage^1,2^. If both the positive and negative voltages are essential to switch resistance state between HRS and LRS, such RS can be termed as bipolar resistive switching (BRS)^2^. On the other hand, if only one polarity of the voltage is sufficient to switch between HRS and LRS, such RS can be termed as unipolar RS (URS). Controlling RS with voltage has been demonstrated by various authors in oxides^3^, nitrides^4^ and organic materials^5^.

On top of that controlling the RS effect with thickness variation has been demonstrated previously. An increase in the reset voltage has been observed by Wang *et al*., upon increase in the thickness of ZnMn_2_O_4_ in Ag/ZnMn_2_O_4_/p^+^-Si based RRAM devices^6^. On the other hand, Zhu *et al*., have reported thickness dependent bipolar resistive switching behavior pertinent to NiO_x_ films, where they observed the clockwise (for low thickness) and anticlockwise (for high thickness) I–V characteristics respectively^7^. Yet in another study, Kang *et al*., have reported that upon increase in the thickness of ZnO in Al/ZnO/Al layered RS memory device, indeed there is an enhancement in the crystallinity of ZnO layer, the concentration of oxygen related defects and set voltages^8^. On the other hand, sharath *et al*., have reported the effect of HfO_2_ layer thickness on the forming voltage. It has been observed that forming voltage increases linearly with HfO_2_ layer thickness^9^. Ito *et al*., have reported on the oxide thickness dependence of resistive switching characteristics for Ni/HfO_*x*_/Pt resistive random access memory device. Their observation has indicated that a clear dependence of switching voltages for the set and reset processes on oxide thickness^10^. Enhanced values of forming voltages have been observed by Inoue *et al*., upon increase in thickness of Fe_2_O_3_ in the Pt/Fe_2_O_3_/Pt based RRAM devices^11^.

Despite the control of RS effect with voltage and thickness of oxide film, the underlying mechanism for the RS effect has been under intense debate. However, two successful models those have been proposed to explain RS mechanism are: (a) Interface model with a redox reaction at the interface between the electrode and the insulating layer (such as modification of Schottky barrier height or width)^12^ and (b) bulk effect or filament model (such as formation of local conductive filaments)^13^.

The crucial parameter to control transport properties/switching mechanism in aforementioned devices is voltage, hinting that only one degree of freedom exists in order to control the switching mechanism. However, recent technologies demand more than one degree of freedom to control switching mechanism. Hence, there is a quest for the manipulation of switching effect with external parameters such as magnetic field, light^14,15^ and temperature^16^. Among them, magnetic fields give an opportunity to control transport/switching mechanism in a remote way. Hence, in the present manuscript, we put our efforts in controlling the transport/switching properties of Ag/TiO_2_/FTO based RRAM device in a remote way using magnetic fields. Earlier, magnetic field control of RS has been well demonstrated on devices which are doped with magnetic elements^17,18^. Bai Sun *et al*.^14^ showed a systematic increase in set and reset voltage of Ag/[BiFeO3/γ-Fe2O3]/FTO device with increasing field strength, which has been explained on the basis of coupling between magnetism and ferroelectricity of BFO. Yet in another study, a delay in transition from HRS to LRS in presence of magnetic field has been well reported by Wang *et al*.^19^ in Si–SiO_2_–MgO device. The resistive switching behavior has been most widely observed in a variety of binary transition metal oxides^20–22^ such as NiO, ZnO, TiO_2_, Nb_2_O_5_ and ZrO_2_.

Recent reports on anatase TiO_2_ in the form of thin film has demonstrated significant magnetic properties^23^. However, magnetic field control of RS in anatase TiO_2_ has not been explored until now. Essentially the magnetic field may create Lorentz force on charge carriers and influence I–V characteristics. Hence, in the present manuscript, we would like to utilize this intriguing phenomena to control RS in anatase TiO_2_ based RRAM devices. Salient features of the present manuscript are (a) controlling the abrupt change of resistance in the low resistance state with a magnetic field (b) tuning the endurance characteristics with magnetic fields (c) attaining multi-bit storage with voltage control and altering it with the magnetic fields.

## Results and Discussion

**Figure 1:**
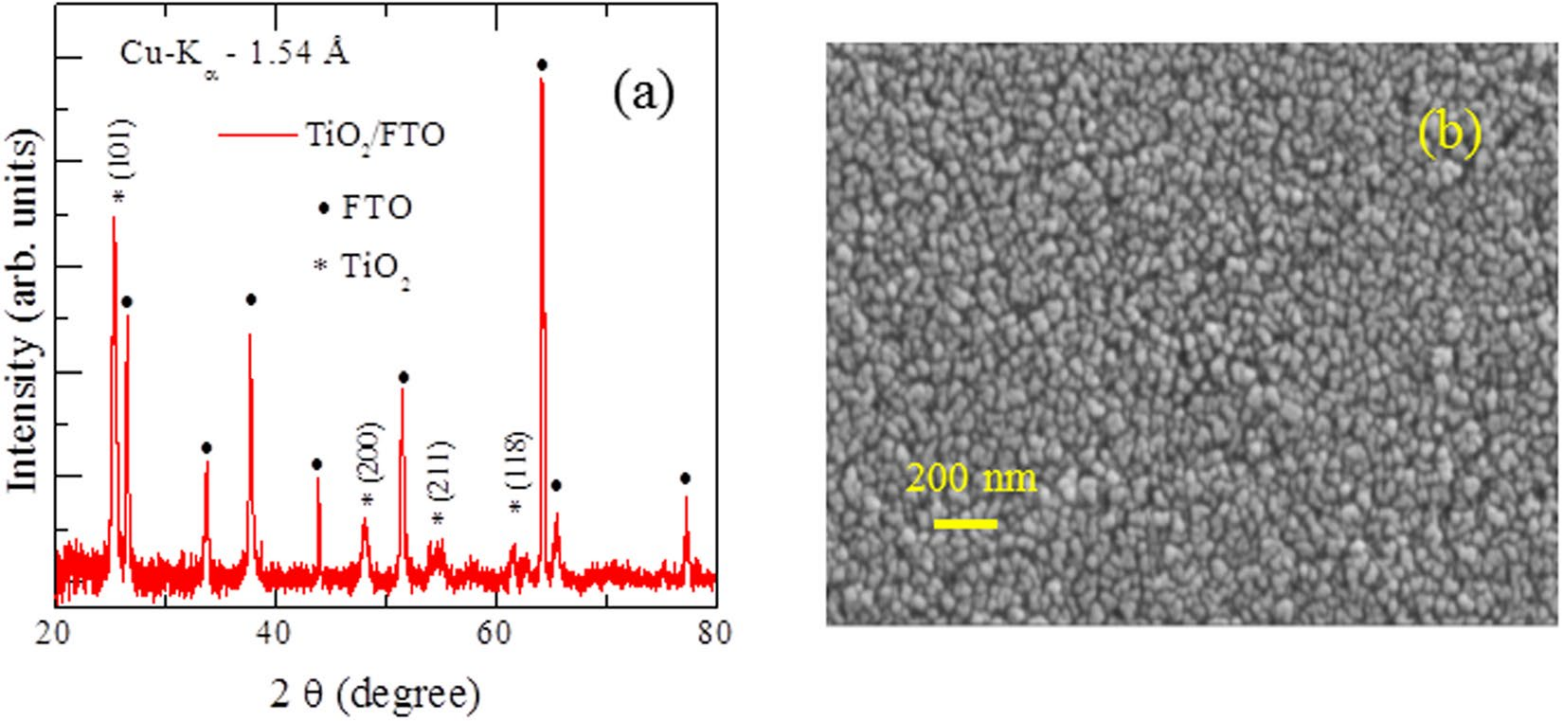
(**a**) Grazing angle XRD pattern of TiO_2_/FTO thin film. It is evident from the figure that in addition to anatase TiO_2_ peaks, peaks from substrate are also present (**b**) FE-SEM image of TiO_2_ thin film. It is clear from the graph that the grain size is 50–60 nm.

Figure 1(a) shows the grazing angle x – ray diffraction pattern obtained from TiO_2_ deposited on FTO substrate (TiO_2_/FTO) after annealing at 400 °C for 1 hour. The diffraction peaks at 25.3° and 47.9° are assigned to (101) and (200) planes respectively, hinting that TiO_2_ is in the anatase phase. All peaks are in line with respect to the standard spectrum pertinent to anatase TiO_2_ (JCPDS no. 84-1286). Apart from TiO_2_ peaks, we also observed peaks from the substrate FTO, which is evident from Fig. 1(a). Figure 1(b) shows the FE-SEM image of TiO_2_ film. It is evident that the grain size is around 50–60 nm. Composition analysis through energy dispersive x – ray spectroscopy (EDX) indicated that Ti and O are in 1:2 phase.

**Figure 2:**
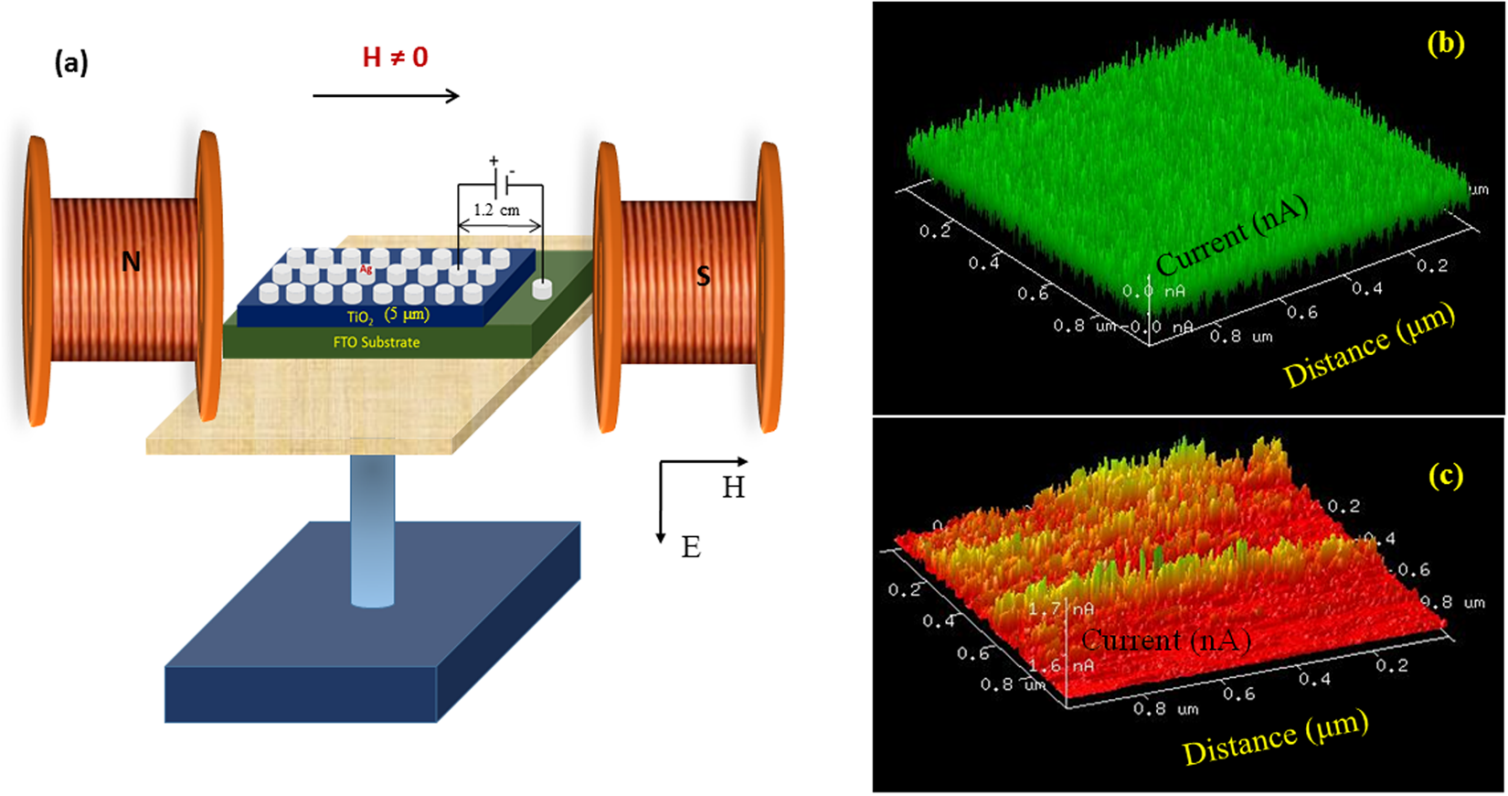
(**a**) Schematic diagram of the measurement performed on TiO_2_ based RRAM device where the magnetic field is applied perpendicular to the direction of the current. Keithley 2400 is used to source the voltage and sense the current respectively (**b**) & (**c**) 3D conducting surface atomic force (C - AFM) images scanned in an area of 1 × 1 µm^2^ by applying a voltage bias of 0 and −8 V respectively.

Figure 2(a) shows the schematic diagram of the experimental setup that we used to perform measurement on TiO_2_ based RRAM device. Figure 2(b) and (c) depicts the 3D C – AFM images from TiO_2_ thin films when the applied bias voltages are 0 V and −8 V respectively. More details about the Fig. 2(a–c) are discussed in methods section.

**Figure 3:**
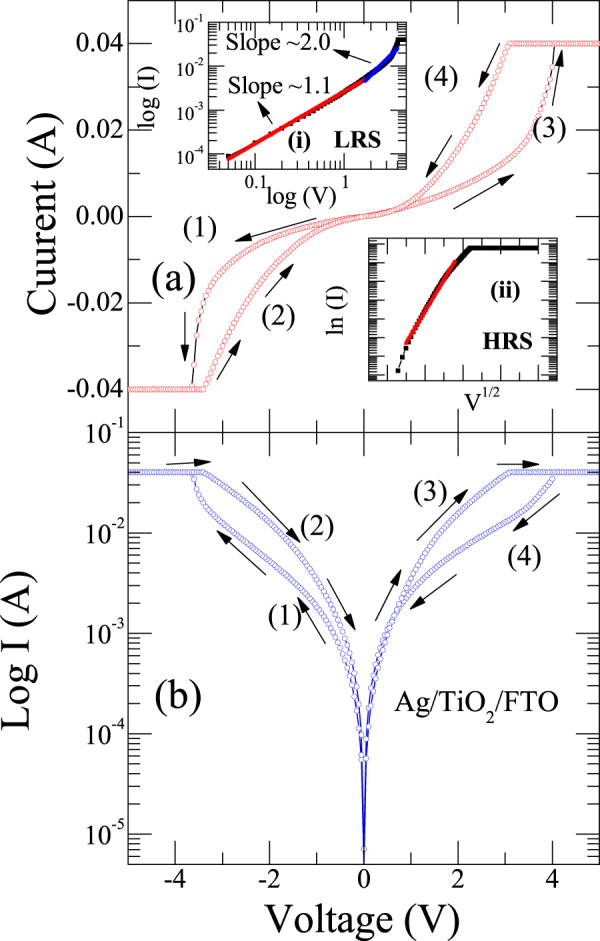
(**a**) The I–V characteristics of the Ag/TiO_2_/FTO device in the range 0 V → −5V → 0 V → +5 V → 0 V. Inset indicates fitting results (i) LRS follows space charge limited conduction mechanism and (ii) HRS follows Schottky emission mechanism (ln I~V^1/2^) (**b**) I~V curve plotted in semi-logarithmic scale.

Figures 3(a) and (b) reveals I–V characteristics of Ag/TiO_2_/FTO device in linear and logarithmic scales respectively. From Fig. 3(a) it is evident that initially, the device is at HRS. By sweeping the voltage from 0 to −5 V with a current compliance of 40 mA (to avoid dielectric breakdown), indeed there is a smooth decrease of current until −3.4 V, above which a sharp jump is evident and device switches to LRS. In the reverse cycle from −5 V to +5 V, the device stayed in LRS and changes to HRS state in the cycle between +5 V to 0 V. This means that in order to switch the device from HRS to LRS (SET switching) (at −3.7 V) or LRS to HRS (RESET switching) (at +3.5 V), we need to apply two different polarities of voltage, which is typical for a BRS. The arrow marks with numbers on the graph shows the sequence that is followed while recording I–V characteristics.

In order to understand the mechanism of resistive switching in our device, we used different conduction models to fit the I–V data. As shown in Fig. 3(a) inset (i), at low voltage region, log (I) *vs*. log (V) shows ohmic conduction behavior (I α V) with a slope of about ~1.1. This indeed followed by a quadratic nature at high voltage regime, which corresponds to Child’s square law (I α V^2^) with a slope of nearly ~2.0. This type of switching behavior from current-voltage relationship can be explained by trap controlled space charge limited current conduction mechanism (SCLC) model where the dielectric layer is triggered by deficiency of oxygen. According to this conduction mechanism, at low voltage region, the thermally generated free charge carrier density inside the film dominates the injected carrier density resulting in an ohmic behavior. When the applied voltage (V) is smaller than the minimum voltage (V_tr_ = voltage required to transit to space charge limited region), the injected carriers redistribute themselves internally due to excess dielectric relaxation time in order to maintain charge neutrality. Concurrently, the probability for injected carriers which can travel across the insulating film is zero. The outset of departure from ohm’s law takes place only when applied voltage reaches V_tr_. Thus, with increasing the applied voltage, the density of injected carriers exceeds the thermally generated carriers to such a value that the Fermi level moves up above the electron trapping level. When all traps are filled, the injected carriers are free to move in the film and excess charges build up in the film, thereby making a transition to SCL conduction, which explains the LRS behavior. Therefore, the generation of conducting path is favored by formation of electron traps and may be due to oxygen vacancies in the TiO_2_ layer results in the change of resistance state pertinent to the device.

On the other hand, in case of HRS, the current behavior follows Schottky emission (SE) mechanism which is verified by ln (I) α V^1/2^ graph as shown in the Fig. 3(a) inset (ii)). The fitting results indicate that due to the thermionic emission between the interface of switching layer and an electrode, electrons cannot climb the potential barrier, which leads less conduction.

**Figure 4:**
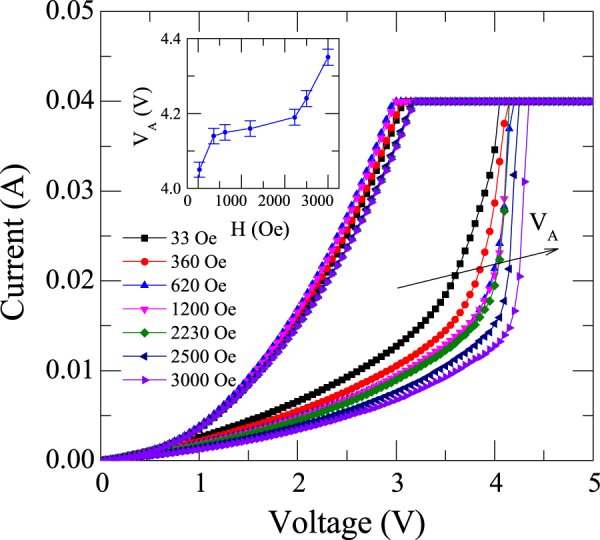
I–V curve of Ag/TiO_2_/FTO device at different magnetic fields. It is evident from the figure that the abrupt voltage (V_A_) shifts to higher voltages with magnetic field due to residual Lorentz force. Inset shows of variation of abrupt voltage (V_A_) with magnetic field.

Now we discuss about the effect of the magnetic field on the BRS in TiO_2_ based RRAM device. As we mentioned in methods section, we applied the external magnetic field parallel to the surface of the film (perpendicular to the current path). From Fig. 3 it is evident that in LRS state there is a sharp change of current from 15 mA to 40 mA around 3.5 V. We define this as an abrupt voltage (V_A_). It is to note that as we apply large amount of magnetic field, high value of V_A_ is required, which manifests the control of transport properties are in a remote way. The evolution related with shift of V_A_ in a remote way is shown in linear plots (Fig. 4). We also performed the measurements when magnetic field is parallel to the current direction. We do not see any shift of V_A_ with H (graph is not shown here as there is no shift). Inset of Fig. 4 depicts the variation of V_A_ with the externally applied transverse magnetic field. The probable mechanism for the variation of V_A_ with the transverse magnetic field can be explained as follows.

Essentially, when a transverse magnetic field is applied with respect to electric field, a Hall field is induced in the device, thereby making the total electric field as a vector sum of applied field and an induced field. Also, under the transverse field, the charge carriers experience a Lorentz force which is thought to be completely cancelled by the induced Hall field only if carriers move with an average drift velocity. If the drift velocity is more than the average drift velocity, the Lorentz force dominates over the force of Hall field resulting in a residual Lorentz force for charge carriers. Mathematically the Lorentz force can be expressed as

$$F=q(E+{\rm{v}}\times {\mu }_{0}H)$$

where *q* is charge of electron, *E* applied electric field, *v* is velocity of electron *μ*_0_ is permeability in free space and *H* externally applied magnetic field. As can be seen from Fig. 2(a), the direction of electric field is from top to bottom and the magnetic field is from left to right. By considering the negative charge on electron the Lorentz force on electron should be out of the paper. Ultimately, a force would influence the motion of electrons due to simultaneous application of electric and magnetic field perpendicular to each other. As a result of this force, electrons would attain cycloid motion, which demands larger value of V_A_ with the magnetic field. Fundamentally, as the magnetic field increases, the force on the electrons increases and hence an increase in V_A_. From this experiment we realize that the residual Lorentz force effect exists only when H is perpendicular to current direction and influences the I–V characteristics of the TiO_2_ based RRAM device.

**Figure 5:**
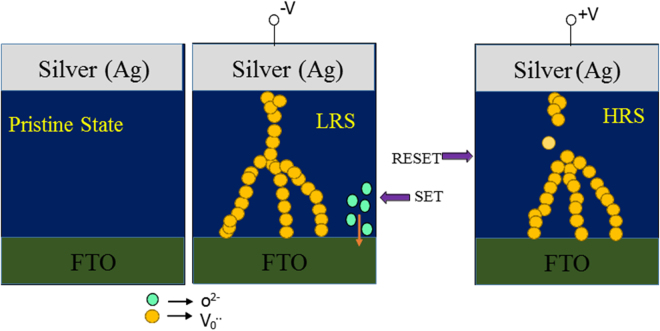
Schematic diagram to explain the bipolar switching mechanism in the absence of magnetic field which corresponds to formation and rupturing of conductive filaments which may be due to electro-migration of oxygen ions.

The conceivable mechanism for the observed RS behavior and filamentary formation can be explained as follows. Figure 5 explains about forming and rupturing of the filaments in Ag/TiO_2_/FTO RRAM device. Essentially, by the application of negative voltage, electro-migration of oxygen ions takes place and may create oxygen vacancies. This indeed leads to formation of filaments between Ag and FTO electrodes, which results in the LRS state. In contrast, for the applied +ve voltage, due to the repulsion between oxygen vacancies and positive charges, rupturing of the filaments takes place, which leads to HRS state.

Earlier, the shift of voltage with the applied magnetic field has been demonstrated in GaAs devices^24^ and correlated such an intriguing phenomena to a residual Lorentz force, shift of Landau level of electrons in conduction band, electron mobility and thermal & impact ionization of electrons^25,26^. Yet in another work, the effect of magnetic field on switching behavior of silicon device also showed the suppression of LRS state confirming that magnetic fields can influence the LRS state^19^. Earlier conductive bridge resistive random access memory (CBRRAM) cell using Ag doped polymer electrolyte between Pt electrodes has well been demonstrated^27^. We rule out this possibility in our current device due to the following reason. (a) we have done extensive analysis of FESEM between two contact Ag pads. We do not see the presence of Ag anywhere between two contact pads upto the depth profile of 1 μm (b) the gap between two Ag contacts is ~1.2 cm.

**Figure 6:**
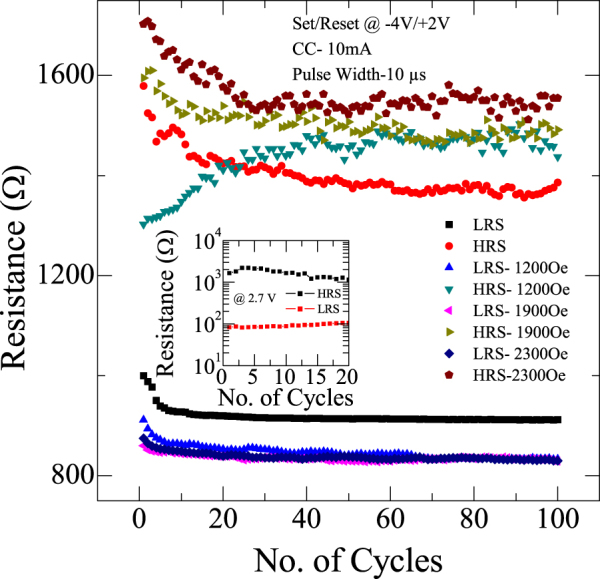
Endurance characteristics of resistive switching for different magnetic fields in the range 0–2300 Oe. It is evident that with magnetic fields, there is an enhancement in the resistance values in LRS and HRS. Inset shows endurance characteristics with a memory window about 10.

Endurance characteristics of TiO_2_ RRAM device are shown in the inset of Fig. 6. Memory window of the cell is calculated using the formula (R_OFF_ − R_ON_)/R_ON_ ≈ R_OFF_/R_ON_ and is found to be 10. Indeed, this window is huge, which essentially makes the device suitable to distinguish the information between 1 and 0. It is also evident that there is no degradation of the device even after repeated cycling which ensure the switching between off and on states is reproducible, reversible and controllable. In order to get more insights on durability of the device, we performed endurance characteristics in the presence of magnetic field using Keithley 4200 semiconductor characterization system. For this purpose, a current compliance of 10 mA and a pulse width of 10 µs with set/reset voltage of −4 V/+2 V is applied to the device. From Fig. 6 it is apparent that the resistance values in both high and low resistance states increase with magnetic field. Such an increase in resistance values with magnetic field can be attributed to Lorentz force as we mentioned earlier.

**Figure 7:**
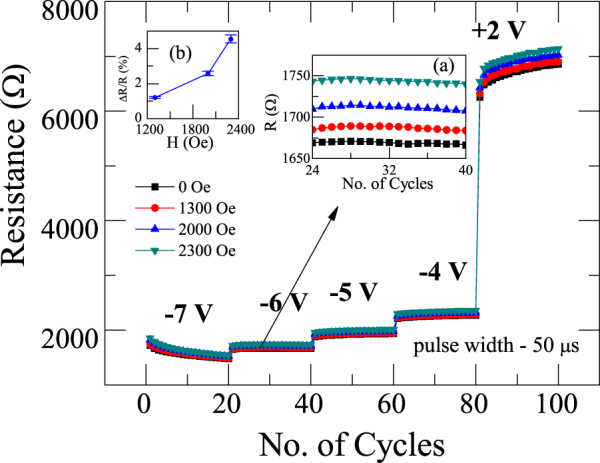
Multilevel resistive switching in Ag/TiO_2_/FTO device at different voltage pulses (−7 V, −6 V, −5 V, −4 V and +2 V) with a width of 50 µs under various magnetic fields in the range 0–2300 Oe.

The endurance characteristics of the device also tested by the application of four different voltage pulses of −7V, −6V, −5V, −4V and +2 V with a pulse width of 50 µs (Fig. 7). Here, negative voltages are applied to the device to set it into different on states and the reset was achieved by applying positive voltage pulse of + 2 V. For example, at −6 V, the device is in low resistance state. It is evident from the inset (a) of Fig. 7 that, there is a systematic increase in the resistance values of the device at various magnetic fields. We also tried to plot the change in resistance (ΔR/R) with respect to zero field value as shown in the inset (b) of Fig. 7. It is evident that nearly 5% change in the resistance value is evident by the application of 2300 Oe, which demonstrates that the resistance state can precisely be tuned in a remote way. Indeed, we could tune resistance state for different on states (at −7 V, −6 V, −5 V and −4 V) as well as for off state (+2 V) as shown in Fig. 7. These results are reproducible and consistent, hinting that tuning of multilevel resistive switching of Ag/TiO_2_/FTO device with the magnetic field in a remote way that promises future application in multi-bit data storage technology.

## Methods

The phase of TiO_2_ was confirmed by (Bruker Discover D8) X-ray diffraction (XRD) with Cu - Kα radiation (λ = 1.54 Å). Microstructural studies were performed using the field emission scanning electron microscope (Zeiss ultra 55 FE-SEM). Composition of the films was determined using the energy dispersive x- ray spectroscopy (EDX) attached with the FE-SEM.

RRAM devices were fabricated on Fluorine doped Tin Oxide (FTO) substrate in order to elucidate the resistive switching behavior in Ag/TiO_2_/FTO device. Initially, TiO_2_ paste (obtained from Solaronix) was used to prepare a thin film on FTO substrate using a drop casting method and subsequently the film was annealed for 1 hr at 400 °C. TiO_2_ layer thickness is around 5 µm. On top of this annealed TiO_2_ films, contacts were given with silver epoxy (~0.3 mm). The distance between two silver (Ag) contacts is nearly 1.2 cm. Here we used Ag as top electrode and FTO as bottom electrode. Ag was used as top electrode because it provides very good conduction. In addition, Ag has commonly been used as an inert electrode material due to its anti-oxidation property. FTO was used as bottom electrode as the work function of FTO and TiO_2_ are close to each other with a difference of 0.4–0.6 eV, we expect the flow of electrons would be easy from FTO to TiO_2_ with a little amount of potential. The resistance of FTO is approximately 10 Ω. Two probe method was used to perform I–V characteristics of Ag/TiO_2_/FTO device at room temperature using a Keithley 2400 meter with a compliance limit of 40 mA. Subsequently, the effect of the magnetic field on the switching behavior was carried out. I–V characteristic of the Ag/TiO_2_/FTO device in the presence of magnetic field was performed in two configurations (a) without magnetic field (H = 0) and (b) with magnetic field (H ≠ 0) (Fig. 2(a)). In the configuration where H ≠ 0, an electromagnet (Walker scientific) was used to generate the magnetic field in the range of 0 to 3000 Oe (Fig. 2(a)). The direction of magnetic field is perpendicular to the motion of electrons in the device (CPP configuration). Keithley 4200 (semiconductor characterization system) was used to perform the endurance characteristics in the presence of magnetic field. Essentialy, the endurance characteristics of the device tested by the application of four different voltage pulses of −7 V, −6 V, −5 V, −4 V and +2 V with a pulse width of 50 µs at various magnetic fields. In order to confirm the role of conductive filaments in the device, we have characterized the device with conductive atomic force microscopy (CAFM). In C-AFM study, the metallic AFM tip behaves as a nano-sized electrode in the switching of the Metal/Insulator/Semiconductor structure. In this method, a voltage was applied between the Pt/Ir coated antimony n - doped Si tip (Commercial Code SCM-PIC) that serves as top electrode and FTO as bottom electrode. The curvature radius, oscillation frequency and cantilever spring constant of the tip are 20 nm, 13 kHz and 0.2 N/m respectively. The experiment was carried out at room temperature and atmospheric pressure without applying any current compliance. We applied bias voltages of 0 V and −8 V in two different experiments on an area of 1.0 × 1.0 µm^2^ at 1.01 Hz in contact mode. When the voltage is 0 V, we do not see any conducting channels in the film (Fig. 2(b)). However, when the bias voltage is −8 V, we do see conducting channels which may be due to oxygen vacancies as shown in Fig. 2(c). This indicates the presence of conducting channels that are distributed across the scanned area. The current mapping image confirms the widely accepted filament model for the resistive switching in TiO_2_.

### Summary

In summary, we demonstrated the stable bipolar resistive switching in Ag/TiO_2_/FTO device and also explored the effect of the magnetic field on the resistance switching behavior. We could tune the abrupt voltage (V_A_) in LRS state remotely and explained on the basis of Lorentz force effect. Endurance characteristics inferred an increase in resistance values with the magnetic field. Tuning the resistance state for different on states (at −7 V, −6 V, −5V and −4 V) as well as for off state (+2 V) with magnetic field reveal that resistance states (both LRS and HRS) can be tuned in a remote way. This indeed hint that the present results may be helpful in future RRAM based devices those would operate with magnetic fields.
